# Aerosol and environmental surface monitoring for SARS-CoV-2 RNA in a designated hospital for severe COVID-19 patients

**DOI:** 10.1017/S0950268820001570

**Published:** 2020-07-14

**Authors:** Y. H. Li, Y. Z. Fan, L. Jiang, H. B. Wang

**Affiliations:** 1 Department of Obstetrics and Gynecology, Union Hospital, Tongji Medical College, Huazhong University of Science and Technology, Wuhan 430022, China; 2 Hospital Infection Control Department, Union Hospital, Tongji Medical College, Huazhong University of Science and Technology, Wuhan 430022, China

**Keywords:** Aerosol, environmental surface, SARS-CoV-2, viral load

## Abstract

There is limited information concerning the viral load of severe acute respiratory syndrome coronavirus 2 (SARS-CoV-2) in aerosols deposited on environmental surfaces and the effectiveness of infection prevention and control procedures on eliminating SARS-CoV-2 contamination in hospital settings. We examined the concentration of SARS-CoV-2 in aerosol samples and on environmental surfaces in a hospital designated for treating severe COVID-19 patients. Aerosol samples were collected by a microbial air sampler, and environmental surfaces were sampled using sterile premoistened swabs at multiple sites. Ninety surface swabs and 135 aerosol samples were collected. Only two swabs, sampled from the inside of a patient's mask, were positive for SARS-CoV-2 RNA. All other swabs and aerosol samples were negative for the virus. Our study indicated that strict implementation of infection prevention and control procedures was highly effective in eliminating aerosol and environmental borne SARS-CoV-2 RNA thereby reducing the risk of cross-infection in hospitals.

## Introduction

Since the outbreak of coronavirus disease 2019 (COVID-19) caused by severe acute respiratory syndrome coronavirus 2 (SARS-CoV-2) at the end of 2019 in Wuhan, China, this severe infection has spread rapidly around the world [1, 2], and was declared a global pandemic by the World Health Organization (WHO) in March 2020 [3]. According to a recent study, 3387 health care workers (HCWs) were infected with COVID-19 in China at the end of February 2020, and more than 20 had died [4]. This pattern of susceptibility of HCWs to infection due to exposure to such patients has been recorded in several countries, with media reports indicating that these workers make up 9% and 15% of Italy's and Spain's COVID-19 cases, respectively. It is therefore imperative to protect HCWs from COVID-19 infection, not only to safeguard continuity of medical services but also to ensure that they do not become prime vectors of transmission [5].

SARS-CoV-2 can spread via respiratory droplets, bodily fluids or contaminated surfaces [6]. Of note, aerosol spread of the earlier SARS-CoV-1 agent, which shares approximately 82% similarity with SARS-CoV-2 [7], appeared to explain the large community outbreak of SARS in Hong Kong in 2003 [8]. Furthermore, a recent report of a cluster of COVID-19 patients presumably due to an asymptomatic infected person in a shopping mall, supports the possibility of indirect transmission of SARS-CoV-2 via surface contamination and/or or aerosols [9]. Indeed, guidelines from the China National Health Commission [10], supports the view of SARS-CoV-2 transmission through aerosols in confined spaces. However, there is limited information on the viral load in aerosols and on environmental surfaces, and the effectiveness of routine nosocomial infection prevention and control procedures on eliminating SARS-CoV-2 contamination in hospitals designated to treat COVID-19 patients due to technical difficulties in collecting viral-laden aerosols and quantifying the virus at low concentrations. It follows that if SARS-CoV-2 is indeed able to survive in aerosols in hospital units treating COVID-19 patients, we may need to revise the current hospital infection prevention and control practices and personal protection strategies.

Currently, a wide variety of aerosol sampling methods are in use and several other methods are in the developmental stage [11]. However, no standard protocol has been available until recently. An impingement air sampler is the most commonly used sampler for collecting aerosolised viruses in which air is drawn in through a narrow inlet tube into an impingement liquid [12]. In this study, we used such a sampler to monitor the viral load in aerosols and swabbing of environmental surfaces in various locations of a designated hospital for treating severe COVID-19 patients in Wuhan, China, the epicentre city of the initial disease outbreak. This served to provide important information for developing nosocomial infection prevention and control measures, and to stem the potential rise of nosocomial cross-transmission of the virus.

## Methods

### Study design

The study was performed from 20 February to 5 March 2020 at Union Hospital, Tongji Medical College, Huazhong University of Science and Technology, which is a designated hospital for treating severe and critical COVID-19 patients diagnosed according to the Chinese management guidelines for COVID-19 (version 7.0) [10]. Patients who had any of the following features were classified as severe cases: (1) respiratory distress (⩾30 breaths per min); (2) oxygen saturation at rest ⩽93%; (3) ratio of partial pressure of arterial oxygen to fractional concentration of oxygen inspired air ⩽300 mm Hg; or (4) severe disease complications (e.g. respiratory failure, required mechanical ventilation, septic shock or non-respiratory organ failure). During the study period, the hospital admitted more than 800 patients, the majority with COVID-19, of whom 20 were treated in the ICU. Infected patients were identified by reverse transcriptase-polymerase chain reaction (RT-PCR) testing for SARS-CoV-2 on admission. Patients in the isolation room were encouraged to wear a triple layer surgical mask at all times. The predetermined environmental surfaces inside and outside the ward were swabbed. Meanwhile, aerosol samples were collected from the ICU ward, general isolation wards, fever clinic, storage room for medical waste, conference rooms and the public area etc. (Supplementary Fig. S1). Heating, ventilation and air conditioning (HVAC) systems were set up according to GB50849-2014 with return air exhausted to the outside or passed through a HEPA filter before circulation. The ICU ward has 12 air inlets with 16 discharges per hour, and the isolation room 8 air inlets with 12 discharges per hour. COVID-19 patients in a ward were separated by a minimum of 1.5 m.

### Sample collection

Approximately 1 h after routine twice-daily cleaning of contact surfaces (using 500 mg/l sodium dichloroisocyanurate) and floors (using 1000 mg/l sodium dichloroisocyanurate) and after 4-time-daily air disinfection using a plasma air steriliser (Laoken Medical Technology Co., Sichuan, China), aerosol samples were collected by an impingement air sampler (BIO-Capturer-6, Bioenrichment Co., Hangzhou, China). This instrument employs the positive potential of SLC-SiOH magnetic beads, which can enrich aerosol particles of diameter 0.03–0.5 μm containing negatively charged RNA viruses [13]. In brief, the air samplers were fixed on a tripod and set at 1.0−1.5 m above the floor level in the wards approximately 1–5 m from the patients' beds for 30 min. A volume of 150 μl magnetic beads was mixed with 45 ml sampling buffer and added to the sampling bottle. A total of 2400 l of air was collected at a rate of approximately 80 l/min per sample. The sampling bottle was placed on a matching magnetic shelf for 5 min to immobilise the magnetic beads, and the sampling buffer was drained off and discarded. Approximately 200 μl of phosphate-buffered saline pH 7.0 with magnetic beads was collected and transferred to a sterile 2 ml tube, transported in an icebox and stored at −80 °C (Supplementary Fig. S2). At each sampling location, three replicate aerosol samples were collected on separate days. Environmental surfaces (*c*. 5 cm^2^) were sampled with saline-moistened swabs after routine cleaning on three separate days. All samples were analysed in the BSL-2 laboratory by RT-PCR in accordance with the WHO protocol [14]. A cycle threshold (Ct) of 40 or greater denoted negative findings for SARS-CoV-2, whereas a Ct of less than 37 denoted positive findings. A Ct of greater than 37 but lower than 40 was considered a suspicious value and was subjected to retesting. SARS-CoV-2 was reported as positive if the second Ct value was less than 40 and an obvious peak was observed or if the second Ct value was less than 37.

## Results

A total of 90 environmental surface swabs was collected from 30 locations inside and outside the isolation wards (Table 1). All, except two, samples from the inside of a COVID-19 patient's mask, were negative for SARS-Co-2 RNA. The two positive samples were taken seven days apart; the patient was in a critical condition and still positive for the virus based on oropharyngeal swabs. A third sample of the patient's mask taken a week later tested negative, as did a concomitant oropharyngeal sample (Table 1). All 135 aerosol samples from 45 locations were negative for SARS-CoV-2 RNA (Table 2).

**Table 1.** SARS-CoV-2 RNA test results from environmental surfaces in a COVID-19 designated hospital

	Sampling locations	Number of samples^a^	Number of positive samples^b^
High-risk area	Bed rails and nightstands in the ICU ward for COVID-19 patients	9	–
	Patients' personal belongings (mobile phone, clothes, pillowcase, towel)	12	–
	Surfaces of medical supplies (infusion pump, operating table in nurse station, temperature gun etc.)	12	–
	Hands of doctor/nurse in the ICU	6	–
	Toilet and sink in isolation ward	6	–
	Door handle in isolation ward	6	–
	Inside of the patient's mask	3	2 (first and second)
	Goggles after use	6	–
Medium-risk area	Door handle in buffer zone	6	–
	Inner wall of waste container	6	–
Low-risk area	Hands of doctor/nurse in clean zone	6	–
	Computer keyboard in nurse station	6	–
	Computer mouse in nurse station	6	
Total		90	2

a All samples were collected 1 h after routine cleaning.

b All samples were tested by qualitative RT-PCR. Sampling and testing were repeated three times at each location.

**Table 2.** SARS-CoV-2 RNA test results for aerosol samples from a COVID-19 designated hospital

	Sampling locations	Number of samples^a^	Number of positive samples^b^
High-risk area	Corridor of ICU ward	9	–
	ICU ward	9	–
	Isolation ward	18	–
	Fever clinic	9	–
	Storage locations for infectious waste	9	–
Medium-risk area	Buffer room in the ICU ward	9	–
	Buffer room in the isolation ward	9	–
Low-risk area	Clean zone in the ICU	9	–
	Clean zone in the isolation ward	18	–
	Public area of the hospital	9	–
	Conference room	18	–
Total		135	–

a All samples were collected after routine cleaning.

b All samples were analysed by qualitative RT-PCR. Sampling and testing were repeated three times at each location.

## Discussion

Consistent with other respiratory acquired viral agents, the main transmission routes of SARS-CoV-2 are via respiratory droplets and close contact. A recent study demonstrated that viable SARS-CoV-2 could be detected in laboratory-generated aerosols up to 3 h post aerosolisation, and the viable virus was detectable up to 4 h on copper, 24 h on cardboard and 2–3 days on plastic and stainless steel [15]. It is therefore valid to speculate that aerosol transmission may occur with prolonged exposure to high concentrations of SARS-CoV-2 aerosol in a relatively closed space [15]. However, more evidence is needed to verify whether airborne transmission of, and environmental surface contamination with, this virus does occur in a hospital setting, as it could pose a serious threat to the safety of HCWs. It follows that monitoring of air and surface contamination due to SARS-CoV-2 plays an important role in developing regulations and guidance for nosocomial infection prevention and control.

In our study, SARS-CoV-2 RNA was not detected in any of 135 aerosol samples from different areas of a designated hospital for severe COVID-19 patients. This result is inconsistent with the findings of Liu *et al*. [16], who reported positive, but low concentrations, of aerosol-borne virus which was reduced to undetectable levels after the implementation of rigorous sanitisation procedures. Three other studies [17–19] also failed to detect SARS-CoV-2 in air samples in wards treating COVID-19 patients. Indeed, in one centre, the virus could not be identified in air samples collected at a distance of 10 cm from the chin of COVID-19 patients not wearing a surgical mask [19]. In contrast, two other studies [20, 21] reported measurable aerosol concentrations of SARS-CoV-2 RNA in isolation or ICU wards, but the viral load was still low; the authors did not report whether the samples were collected before or after cleaning. Further research is clearly needed to determine whether inconsistent findings between studies are related to the different air sampler used, the flow rate and the duration of aerosol sampling. Nevertheless, our results suggest that when strict disinfection procedures are implemented and room ventilation is maintained, the likelihood of aerosol-borne SARS-CoV-2 in the hospital must be considered to be low.

Furthermore, with the exception of two samples from a COVID-19 patient's mask, all 90 environmental surfaces sampled were negative for SARS-CoV-2. Although viral RNA was detected by Ong *et al*. [17] on some environmental surfaces and personal protective equipment (PPE) in isolation rooms (air outlet fans, toilet sites) for COVID-19 patients, all samples proved negative after routine cleaning. Two other recent studies from China [22] and Italy [23] also tested hospital environmental surfaces, or PPE, of staff members for SARS-CoV-2 RNA but all failed to detect the virus. In contrast, several other studies also measured surface concentrations of viral RNA in the ICU and isolation wards [20, 21, 24, 25] and in a quarantine hotel [25], and reported widespread surface contamination with SARS-CoV-2, but all failed to specify whether sampling was conducted before or after cleaning. Moreover, two studies were performed in a relatively closed temporary hospital or in rooms that may have lacked adequate disinfection and ventilation [20, 25].

Based on our evidence, we believe that the current disinfection measures used in our hospital setting are sufficient to eliminate, or reduce to undetectable levels, SARS-CoV-2 contamination. Since the end of January 2020, to date, our hospital has admitted more than 1600 patients and none of 3255 HCWs in the hospital has become infected. Nevertheless, the detection of viral RNA on the mask worn by the COVID-19 patient reminds us to remain cautious and vigilant during close contact with patients with strict adherence to hand hygiene. From our experience, we propose that the following measures are essential for achieving a safe hospital environment during the COVID-19 epidemic. (i) An isolation ward should be set up with ‘three zones and two channels’, namely, *clean*, *buffer* and *contaminated* zones, with *doctor* and *patient* channels. The isolation ward should have negative pressure ventilation with 12 or more air changes per hour. (ii) Strict policies should be in place for environmental disinfection/cleaning and hand hygiene [26]. (iii) Periodic monitoring of air and environmental surfaces for viral and bacterial load must be conducted, and based on these results, strategies for nosocomial infection prevention and control should be promptly adjusted. (iv) All staff, including HCWs, maintenance workers, food suppliers etc., must be well trained on hand hygiene, respiratory etiquette, donning/removing and proper disposal of PPEs and biomedical waste management. A hospital infection-control team must be assigned to supervise and ensure that all requirements and regulations are being correctly implemented by the medical staff. Finally, in our hospital, we suggest that all patients in isolation rooms should wear a triple-layer surgical mask at all times. This measure may be related to our low detection rates of SARS-CoV-2 RNA in aerosols and on environmental surfaces [27].

There are several limitations to this study. First, the volume of air sampled and the number of environmental surfaces swabbed represent only a small fraction of the whole; thus, some contaminated areas may have been missed. Second, a qualitative test of SARS-Co-2 RNA was used, and therefore we cannot rule out the presence of low concentration of virus below the detection threshold in these samples. Lastly, because viral culture was not performed, we are unable to confirm the presence of viable virus on the environmental surfaces sampled.

In conclusion, our findings suggest that the current infection prevention control practices utilised in a designated COVID-19 hospital appear to be very effective in reducing and lowering the risk of SARS-CoV-2 transmission from aerosols and environmental surfaces to other patients and HCWs.

## Data Availability

The data that support the findings of this study are available from the corresponding author, HW, upon reasonable request.

## Supplementary material

For supplementary material accompanying this paper visit http://dx.doi.org/10.1017/S0950268820001570. click here to view supplementary material

## References

[1] Kinross P (2020) Rapidly increasing cumulative incidence of coronavirus disease (COVID-19) in the European Union/European Economic Area and the United Kingdom, 1 January to 15 March 2020. Euro Surveillance 25, 2000285. 10.2807/1560-7917.ES.2020.25.11.2000285 PMC7096777 32186277

[2] Chen N (2020) Epidemiological and clinical characteristics of 99 cases of 2019 novel coronavirus pneumonia in Wuhan, China: a descriptive study. Lancet (London, England) 395, 507–513. 10.1016/S0140-6736(20)30211-7 PMC7135076 32007143

[3] Cucinotta D and Vanelli M (2020) WHO Declares COVID-19 a pandemic. Acta Bio-medica: Atenei Parmensis 91, 157–160. 32191675 10.23750/abm.v91i1.9397 PMC7569573

[4] Team NCPERE (2020) [The epidemiological characteristics of an outbreak of 2019 novel coronavirus diseases (COVID-19) in China]. Zhonghua liu xing bing xue za zhi=Zhonghua liuxingbingxue zazhi 41, 145–151. 32064853 10.3760/cma.j.issn.0254-6450.2020.02.003

[5] Chang D (2020) Protecting health-care workers from subclinical coronavirus infection. Lancet Respiratory Medicine 8, e13. 32061333 10.1016/S2213-2600(20)30066-7 PMC7128440

[6] Chan JF-W (2020) A familial cluster of pneumonia associated with the 2019 novel coronavirus indicating person-to-person transmission: a study of a family cluster. Lancet (London, England) 395, 514–523. 10.1016/S0140-6736(20)30154-9 PMC7159286 31986261

[7] Chan JF-W (2020) Genomic characterization of the 2019 novel human-pathogenic coronavirus isolated from a patient with atypical pneumonia after visiting Wuhan. Emerging Microbes & Infections 9, 221–236. 31987001 10.1080/22221751.2020.1719902 PMC7067204

[8] Yu IT (2004) Evidence of airborne transmission of the severe acute respiratory syndrome virus. New England Journal of Medicine 350, 1731–1739. 15102999 10.1056/NEJMoa032867

[9] Cai J (2020) Indirect virus transmission in cluster of COVID-19 cases, Wenzhou, China, 2020. Emerging Infectious Diseases 26, 1343–1345. 32163030 10.3201/eid2606.200412 PMC7258486

[10] National Health Commission of the People's Republic of China. Guidelines for the diagnosis and treatment of coronavirus disease 2019 (trial version 7). http://www.nhc.gov.cn/yzygj/s7653p/202003/46c9294a7dfe4cef80dc7f5912eb1989.shtml (Accessed 13 March 2020).

[11] Yoo K (2017) Molecular approaches for the detection and monitoring of microbial communities in bioaerosols: a review. Journal of Environmental Sciences 51, 234–247. 10.1016/j.jes.2016.07.002 28115135

[12] Pan M, Lednicky JA and Wu CY (2019) Collection, particle sizing and detection of airborne viruses. Journal of Applied Microbiology 127, 1596–1611. 30974505 10.1111/jam.14278 PMC7167052

[13] Zhao X (2019) Airborne transmission of influenza virus in a hospital of Qinhuangdao during 2017–2018 flu season. Food and Environmental Virology 11, 427–439. 31549297 10.1007/s12560-019-09404-1

[14] World Health Organization. Clinical management of severe acute respiratory infection when novel coronavirus (2019-nCoV) infection is suspected: interim guidance. https://apps.who.int/iris/handle/10665/330893 (Accessed 28 January 2020).

[15] van Doremalen N (2020) Aerosol and surface stability of SARS-CoV-2 as compared with SARS-CoV-1. New England Journal of Medicine 382, 1564–1567. 32182409 10.1056/NEJMc2004973 PMC7121658

[16] Liu Y (2020) Aerodynamic analysis of SARS-CoV-2 in two Wuhan hospitals. Nature 582, 557–560. 32340022 10.1038/s41586-020-2271-3

[17] Ong SWX (2020) Air, surface environmental, and personal protective equipment contamination by severe acute respiratory syndrome coronavirus 2 (SARS-CoV-2) from a symptomatic patient. JAMA 323, 1610–1612. 10.1001/jama.2020.3227 PMC7057172 32129805

[18] Faridi S (2020) A field indoor air measurement of SARS-CoV-2 in the patient rooms of the largest hospital in Iran. Science of the Total Environment 725, 138401. 32283308 10.1016/j.scitotenv.2020.138401 PMC7194859

[19] Cheng VCC (2020) Escalating infection control response to the rapidly evolving epidemiology of the coronavirus disease 2019 (COVID-19) due to SARS-CoV-2 in Hong Kong. Infection Control and Hospital Epidemiology 41, 493–498. 32131908 10.1017/ice.2020.58 PMC7137535

[20] Guo ZD (2020) Aerosol and surface distribution of severe acute respiratory syndrome coronavirus 2 in hospital wards, Wuhan, China, 2020. Emerging Infectious Diseases 26, 1583–1591. 32275497 10.3201/eid2607.200885 PMC7323510

[21] Chia PY (2020) Detection of air and surface contamination by SARS-CoV-2 in hospital rooms of infected patients. Nature Communications 11, 2800. 10.1038/s41467-020-16670-2 PMC7260225 32472043

[22] Wang J (2020) SARS-CoV-2 RNA detection of hospital isolation wards hygiene monitoring during the coronavirus disease 2019 outbreak in a Chinese hospital. International Journal of Infectious Diseases 94, 103–106. 32311449 10.1016/j.ijid.2020.04.024 PMC7165090

[23] Colaneri M (2020) Lack of SARS-CoV-2 RNA environmental contamination in a tertiary referral hospital for infectious diseases in Northern Italy. Journal of Hospital Infection 105, 474–476. 10.1016/j.jhin.2020.03.018 PMC7156210 32201338

[24] Wu S (2020) Environmental contamination by SARS-CoV-2 in a designated hospital for coronavirus disease 2019. American Journal of Infection Control S0196–6553, 30275–30273. 10.1016/j.ajic.2020.05.003 PMC7214329 32407826

[25] Jiang FC Detection of severe acute respiratory syndrome coronavirus 2 RNA on surfaces in quarantine rooms. Emerging Infectious Diseases. Published online: 18 May 2020. doi: 10.3201/eid2609.201435. PMC7454114 32421495

[26] Weber DJ (2010) Role of hospital surfaces in the transmission of emerging health care-associated pathogens: norovirus, *Clostridium difficile*, and *Acinetobacter* species. American Journal of Infection Control 38(Suppl 1), S25–S33. 20569853 10.1016/j.ajic.2010.04.196

[27] Nardell EA, Nathavitharana RR. Airborne spread of SARS-CoV-2 and a potential role for air disinfection. JAMA. Published online: 1 June 2020. doi: 10.1001/jama.2020.7603. 32478797

