# Supplementary figures and images for: Aerosol and environmental surface monitoring for SARS-CoV-2 RNA in a designated hospital for severe COVID-19 patients
Source: Epidemiol Infect. 2020 Jul 14;148:e154. doi: 10.1017/S0950268820001570 (PMC7371847; doi:10.1017/S0950268820001570)

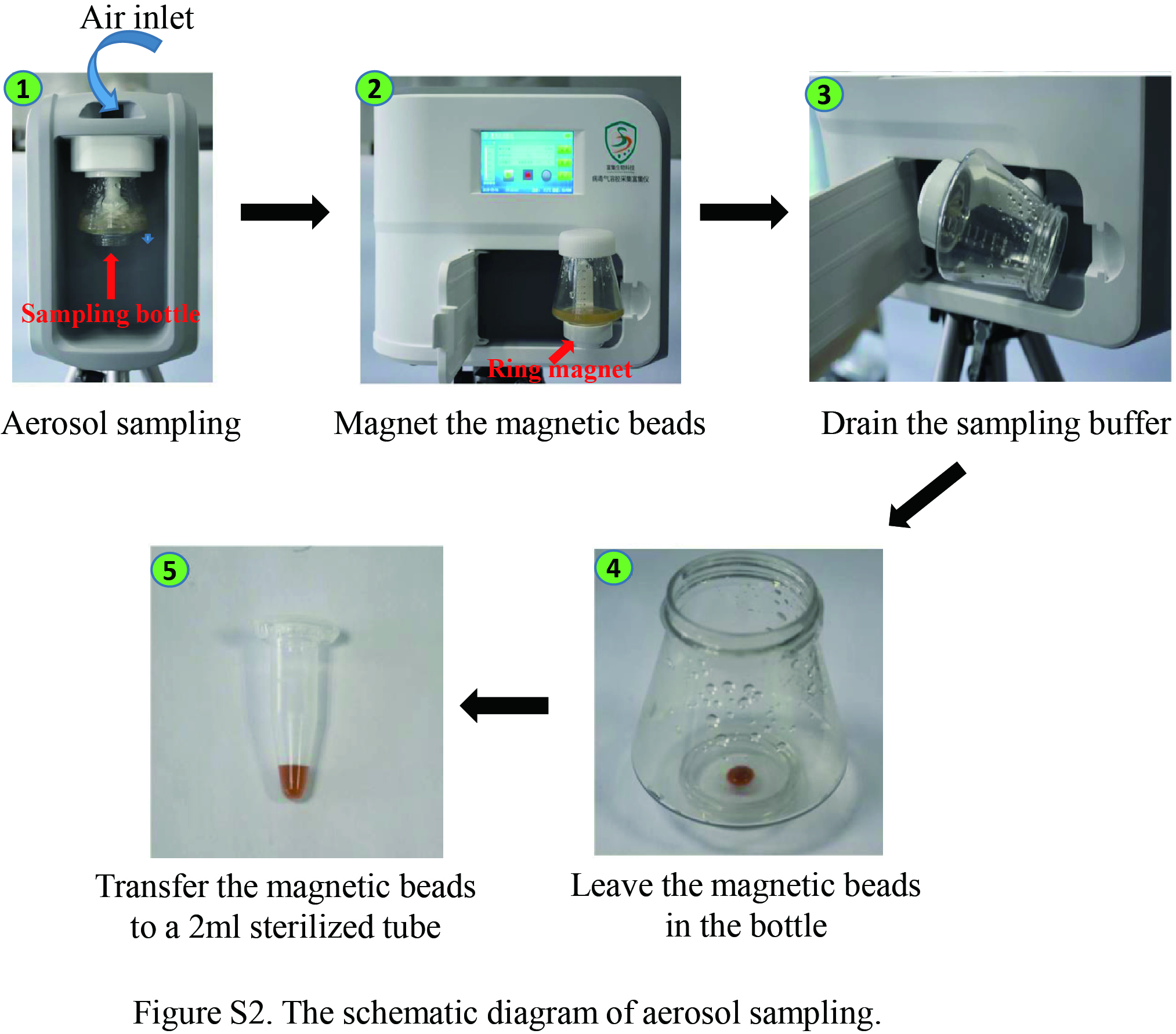

Supplement: Supplementary file 1 [file S0950268820001570sup.zip › S0950268820001570sup001.jpg]

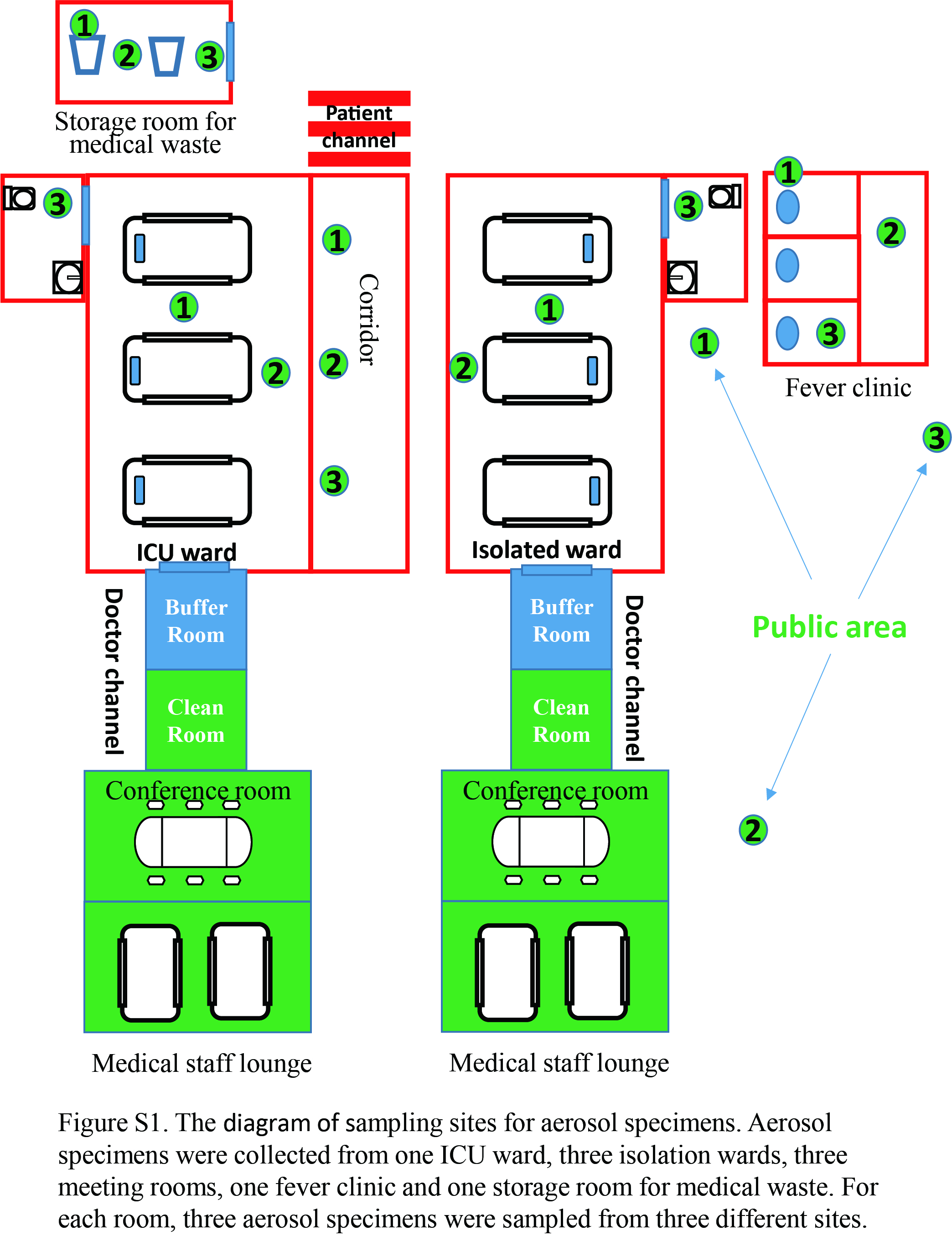

Supplement: Supplementary file 1 [file S0950268820001570sup.zip › S0950268820001570sup002.jpg]
